# Design of Multifunctional Janus Metasurface Based on Subwavelength Grating

**DOI:** 10.3390/nano11041034

**Published:** 2021-04-19

**Authors:** Ruonan Ji, Chuan Jin, Kun Song, Shao-Wei Wang, Xiaopeng Zhao

**Affiliations:** 1 Smart Materials Lab, School of Physical Science and Technology, Northwestern Polytechnical University, Xi’an 710129, China; songkun@nwpu.edu.cn (K.S.); xpzhao@nwpu.edu.cn (X.Z.); 2 State Key laboratory of Transient Optics and Photonics, Xi’an Institute of Optics and Precision Mechanics of CAS, Xi’an 710119, China; jinchuan@opt.ac.cn; 3 State Key Laboratory for Infrared Physics, Shanghai Institute of Technical Physics, Chinese Academy of Sciences, Shanghai 200083, China

**Keywords:** metasurface, polarization, deflector, lens

## Abstract

In this paper, a Janus metasurface is designed by breaking the structural symmetry based on the polarization selection property of subwavelength grating. The structure comprises three layers: a top layer having a metallic nanostructure, a dielectric spacer, and a bottom layer having subwavelength grating. For a forward incidence, the metal-insulator-metal (MIM) structure operates as a gap plasmonic cavity if the linearly polarized (LP) component is parallel to the grating wires. It also acts as a high-efficiency dual-layer grating polarizer for the orthogonal LP component. For the backward incidence, the high reflectance of the grating blocks the function of the gap plasmonic cavity, leading to its pure functioning as a polarizer. A bifunctional Janus metasurface for 45 degrees beam deflector and polarizer, with a transmission of 0.87 and extinction ratio of 3840, is designed at 1.55 μm and is investigated to prove the validity of the proposed strategy. Moreover, the proposed metasurface can be cascaded to achieve more flexible functions since these functions are independent in terms of operational mechanism and structural parameters. A trifunctional Janus metasurface that acts as a focusing lens, as a reflector, and as a polarizer is designed based on this strategy. The proposed metasurface and the design strategy provide convenience and flexibility in the design of multifunctional, miniaturized, and integrated optical components for polarization-related analysis and for detection systems.

## 1. Introduction

Metasurface is a planar array, composed of subwavelength artificial meta-atoms. Owing to its ultrathin thickness, ease of integration, and efficient manipulation of the electromagnetic wave’s amplitude, phase, and polarization, it has been a hot topic of research in the field of photonics [1,2,3]. In addition, it also provides a promising solution for achieving ultrathin, integrable, and multifunctional optical elements [4]. These elements are essential for some applications that require small size, lightweight, and stable optical systems. Multifunctional metasurfaces can be roughly divided into two categories—the dynamic scheme, and the static scheme. Therein, in the dynamic scheme, function switching is obtained by integrating the phase-change materials (e.g., GeSbTe or VO_2_) [5,6] or metal-dielectric phase transition [7,8]; in the static scheme, the switching capabilities are related to different polarizations or propagation directions of light. However, the previous literature mainly focused on the polarization switching of the single transmission or reflection mode [7,9,10,11,12], while the function switching for different propagation directions has been rarely exploited.

Janus is the name of the two-faced God in Roman mythology. In 1992, Gennes first proposed the concept of a special kind of particles with inherent out-of-plane asymmetry that combines dual physical and chemical properties, naming them as Janus particles, after the Roman God [13]. The concept of the Janus particle opened a new chapter for scientific research, with potential applications in the fields of catalysis [14], chemistry [15], quantum optics [16], and biomedicine [17]. Recently, the concept of Janus particles has also been applied for the design of bifunctional metasurface, whose function varies with the propagation direction by breaking the out-of-plane structural symmetry. For example, Yang et al. reported a three-dimensional (3D) Janus metamaterial composed of two interlaced plasmonic three-dimensional helical nanoaperture enantiomers for achieving direction-controlled polarization-encrypted data storage [18]. Comparing with 3D metamaterials, planer metasurfaces are easier for fabrication, and the fabrication process is more compatible with the common complementary metal-oxide semiconductor (CMOS) preparation process. Recently, Chen et al. proposed a five-layer Janus metasurface comprising three cascaded and twisted anisotropic square split-ring resonators to achieve spatial asymmetric wavefront shaping for the single LP component [19]. However, the orthogonal polarization component was always blocked, leading to a fundamental restriction of low energy efficiency.

In this paper, a trilayer MIM Janus metasurface is proposed based on the polarization selection property of the subwavelength grating. The structure symmetry was breaking to achieve different functions by switching the propagation direction or polarization state of the incident light. Moreover, the realization of functions for different polarization states is based on different working mechanisms and different dominated parameters; therefore, mutual influence can be avoided. A bifunctional Janus metasurface that can act as a polarizer and as a deflector was demonstrated to prove the validity of the proposed metasurface. In addition, the proposed unit cells can be combined to achieve more flexible functions. A trifunctional Janus metasurface that acts as a focusing lens, as a beam deflector, and as a polarizer is demonstrated as an example.

## 2. Unit Cell Design

**Figure 1:**
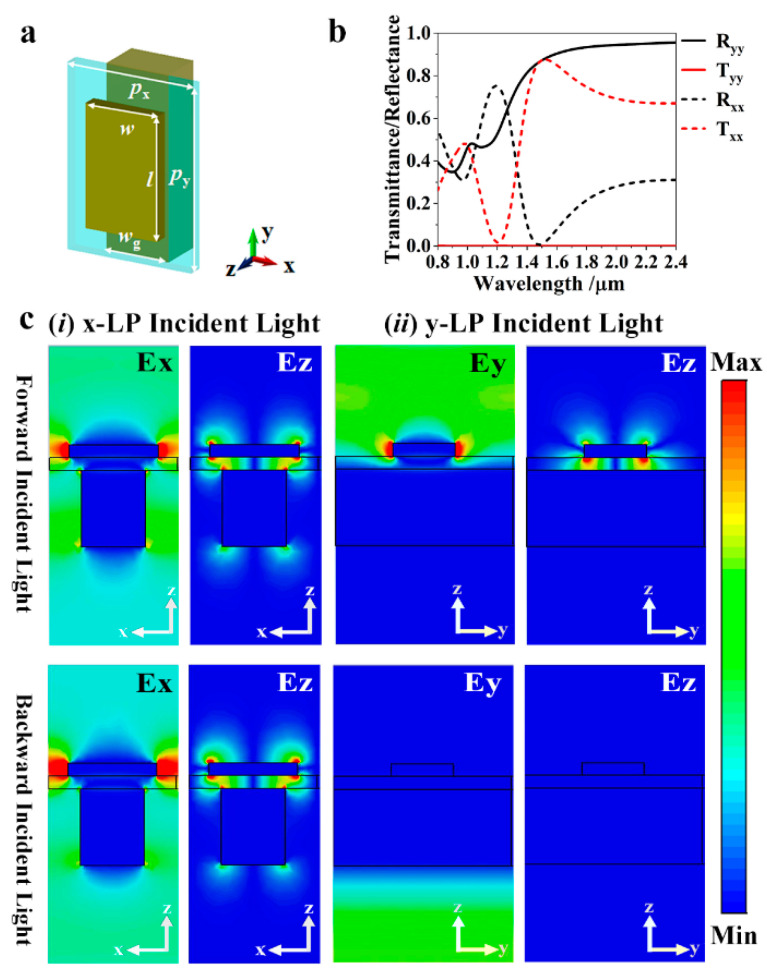
(**a**) Schematic diagram of the proposed Janus metasurface’s unit cell. (**b**) Transmittance and reflectance for *x*- and *y*-LP incidences. (**c**) The unit cell’s electric field distribution for (i) *x*- and (ii) *y*-LP incidences at 1530 nm from forward and backward directions. For these simulations, the structure parameters were taken as *p_x_* = 600 nm, *p_y_* = 432 nm, *w* = 300 nm, *w_g_* = 216 nm, *l* = 210 nm.

Figure 1a shows the unit cell of the proposed Janus metasurface. It consists of three layers: a 50 nm thick top metallic nanostructure, a 50 nm thick dielectric spacer, and a bottom subwavelength grating having a thickness of 300 nm. The bottom subwavelength wire grating acts as a broadband mirror for the polarization state parallel to the grating wires. It couples the surface plasmon polariton wave to achieve extraordinary transmission for the orthogonal polarization state. The top nanostructure is responsible for achieving the polarization-dependent response of the proposed Janus metasurface. Here, a metallic wire structure is taken as an example. The optical properties of the metasurface composed of uniform unit cells were simulated using the finite element method. Periodical boundary conditions were applied in the *x* and the *y* directions. Wave-guide ports were applied in *z* direction. Aluminum was chosen as the metal for the top and bottom layers, and its permittivity is taken from the handbook of Palik [20]. Silicon dioxide (SiO_2_) with a refractive index of 1.5 was taken as the dielectric spacer.

**Figure 2:**
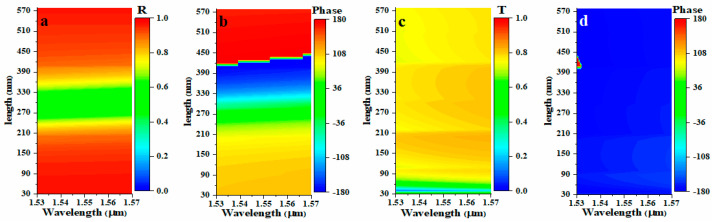
(**a**) The reflection for *y*-LP incidence, (**b**) the phase for *y*-LP incidence, (**c**) transmission for *x*-LP incidence, and (**d**) phase for *x*-LP incidence as a function of the wire length. The parameters are the same as shown in Figure 1, but the length of the wire is varied.

As shown in Figure 1b, with the direction of propagation in the positive *z* direction, the transmission of *y*-LP (i.e., polarization parallel to the grating wire) are totally blocked by the subwavelength grating, while the transmission of *x*–LP (i.e., polarization orthogonal to the grating wire) exhibit a transmission peak at about 1530 nm. To reveal the mechanism, the electric field distributions under *x*- and *y*-LP incident light at 1530 nm were simulated. As shown in Figure 1c(i), the high transmission of *x*-LP originates from the coupling of the localized surface plasmon (LSP) and surface plasmon polariton (SPP) waves, respectively, excited at the surface of the top wire and bottom grating. Therefore, the wavelength of transmission peak nearly remains unchanged when the length of the top wire increase as it is mainly dominated by the widths of the top wire and the grating (see Figure S1a in Supplementary Materials). Moreover, the almost identical electric field distribution can be observed for the *x*-LP incident light propagating in the backward direction (*z* negative), indicating the same transmission property as the forward propagation one. With the forward propagating *y*-LP light, as shown in Figure 1c(ii), the top wire, the spacer, and the grating act as a gap surface plasmon (GSP) resonant cavity [21,22]. The incident light excites the electric dipole along the length of the wire on the top layer, contributing to a resonance corresponding to the wire length [22]. Thus, when the length of the wire increases, the resonant wavelength increases linearly as shown in Figure S1b in Supplementary Materials. Although the resonance also causes a resonant absorption, the reflectance near the resonant wavelengths is above 0.45, and its value at the nonresonant wavelengths can exceed 0.9 in the range of 1000–2000 nm. The resonance happens near 1550 nm corresponding to the wire length of 300 nm (see Figure 2a). The occurrence of resonance brings about a significant phase change, as shown in Figure 2b. A phase shift covering 0°–360° can be achieved by changing the wire length from 90 nm to 450 nm. As for backward propagated *y*-LP incident light, the high reflection of the grating blocks the interaction of the top structure and the orthogonal polarization component to act as an efficient polarizer. Therefore, the out-of-plane structure symmetry under *y*-LP incident light is breaking to achieve a Janus metasurface. Here, one should notice that the metallic wire structure can be replaced by other nanostructures having polarization conversion function, for example, split rings, V-shaped structures, and S-shaped structures to achieve more interesting functions, such as optical activity and linear-circular polarization conversion. In addition, the following design for wavefront shaping at 1550 nm is based on the large and dispersive propagation shifts near the resonant wavelengths; therefore, even considering the tolerance for the phase deviation of the metasurface, the operation bandwidth is still limited. However, the operating wavelength can be shifted, in principle, to the visible or mid-infrared wavelength ranges just by scaling the structural parameters. Due to the dispersion of the material and the current fabrication limitation, usually, the dimension of the structure should be larger than 50 nm, and further parameter optimization is necessary. More importantly, the proposed strategy is available for broadband applications, only need to design a GSP cavity with low Q to obtain quasi-nondispersive phase modulation [22,23].

## 3. Janus Metasurface Based on Subwavelength Gratings

### 3.1. Bifunctional Janus Metasurface as a Polarizer and Deflector

In practical applications, metallic subwavelength gratings are the common linear polarizers, used for optical communication, detection, and other optical systems. Due to the working principle mentioned above, the unselected polarized light will be reflected by the grating, adding noise to the optical system, especially the laser-involved systems. Therefore, the linear polarizer that can transmit the selected polarized light and reflect the unselected light is highly desired. Based on the proposed unit cells, a sandwich-shaped Janus metasurface that implements the functionality of a polarizer and of a beam deflector at 1550 nm is designed in response to satisfy this requirement.

To transmit the *x*-LP component, and to deflect the *y*-LP component with reflection angle of 45°, a supercell composed of five unit-cells is designed. According to the generalized Snell’s law, for an incident plane wave reflected at the interface between two media (the refractive indices are, respectively, *n_i_* and *n*), the angle of incidence *θ_i_* and angle of reflection *θ_r_* satisfy the following equation [24].

(1) $$\sin\theta_{r}-\sin\theta_{i}=\frac{\lambda }{2\pi n_{i}}\frac{d\varphi }{dx}$$

where *λ* is the incident wavelength, and *dφ*/*dx* is the phase gradient introduced along the interface between the two media. As for metasurface with a phase discontinuity, when the plane wave is normally incident (*θ_i_* = 0), the media above the metasurface is air (*n_i_* = 1), and the period of the unit cells composed of the metasurface is Δ*x*, the constant gradient of phase discontinuity Δ*φ* can be expressed as

(2) $$\Delta \varphi =\frac{2\pi }{\lambda }\Delta x\sin\theta_{r}$$

**Figure 3:**
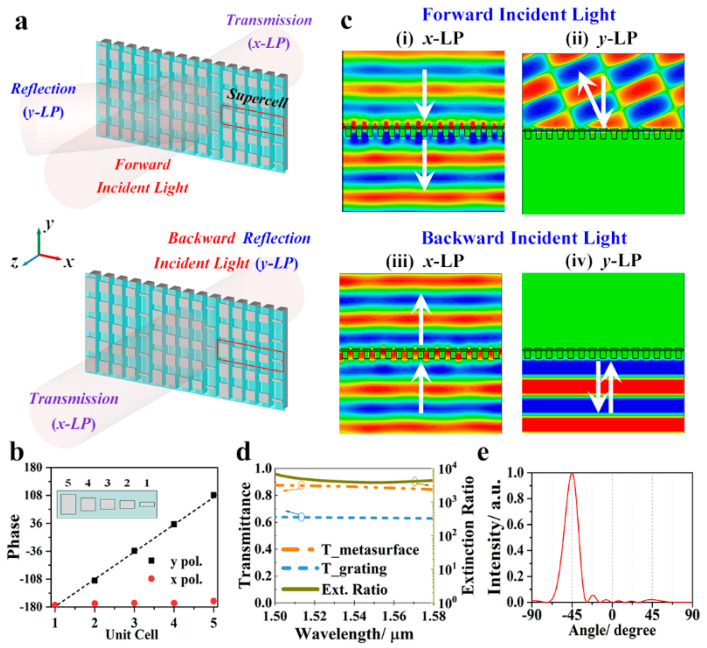
(**a**) Perspective view of the proposed bifunctional Janus metasurface composed of five phase-gradient subunit cells. The metasurface deflects *y*-LP reflection and transmits *x*-LP component for forward incidence. It reflects the *y*-LP component and transmits the *x*-LP component for backward incidence. The geometrical parameters are the same as shown in Figure 1, except that the lengths of the top wires are *l_1_* = 420 nm, *l_2_* = 330 nm, *l_3_* = 290 nm, *l_4_* = 260 nm, *l_5_* = 60 nm. (**b**) The values of the phase for the five subunit cells for *x*–LP and *y*–LP incidences from z positive direction. (**c**) The electric field distribution for forward incident (i) *x*-LP and (ii) *y*-LP and for backward incident (iii) *x*-LP and (iv) *y*-LP waves. (**d**) The transmittance curves the proposed metasurface with a single grating for *x*-LP wave. (**e**) The far-field distribution of the forward incident *y*-LP light.

According to Equation (2), when *λ* = 1.55 μm, Δ*x = p_x_ =* 432 nm, *θ_r_* = 45°, the calculated constant gradient of phase discontinuity Δ*φ* is 72°. Therefore, the lengths of the top wires are optimized as *l_1_* = 420 nm, *l_2_* = 330 nm, *l_3_* = 290 nm, *l_4_* = 260 nm, *l_5_* = 60 nm to achieve a phase gradient of 72°, as shown in Figure 3a,b. The distribution of the electric fields, the transmission, and far-field distribution were obtained using the same simulation method as that used for the metasurface composed of uniform unit cells. As shown in Figure 3c, whether the *x*-LP plane waves were incident normally from the forward or from the backward direction, normal plane wave transmissions are to be observed. In addition, the related transmittance curves are the same. For the *y*-LP plane wave, when it is incident from the forward direction, an abnormal reflection occurs. The far-field analysis shows that the reflection angle is 45°, which is consistent with the theoretical results. As for the backward incident *y*-LP wave, the plane wave is reflected by the grating. In summary, for forward incidence, the proposed metasurface operates as a polarization filter for *x* polarization and as a 45° deflector for the y polarization (see Figure 3e). Comparing with the previously reported bifunctional metasurfaces [10,11,25], the utilization of pixels is effectively improved since the polarization-dependent function switching is based on different working mechanisms, rather than different sets of antennas. For the backward incidence, as shown in Figure 3d, it acts as a polarizer with a transmission of 0.86 at 1.55 μm, which is 36.5% higher than that of the single grating (the transmission is 0.63 at 1.55 μm). In addition, the extinction ratio (T_x_/T_y_) can reach 3840, and this performance can be improved further by optimizing the performance of the composed unit cells.

### 3.2. Multifunctional Janus Metasurface as a Polarizer, Lens, and Deflector

As mentioned above, the modulation performance of the metasurface for the *x*-LP and *y*–LP components are, respectively, based on enhanced transmission by the surface plasmon polariton coupling and the gap plasmonic resonance. Moreover, these two effects depend on the width and the length of the top wire, respectively. Thus, we can cascade two Janus metasurfaces to integrate more functions. Here, a trifunctional Janus metasurface with *y*-LP focusing (under forward incidence), *y*-LP beam deflecting (under backward incidence), and polarization filtering of *x*-LP component is presented as an example.

**Figure 4:**
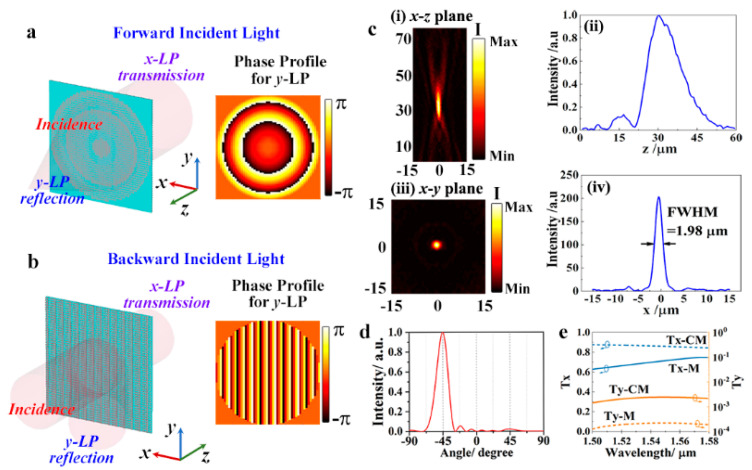
Schematic diagram for the functionalities of the CM for (**a**) forward and (**b**) backward incidences and the phase profile for the *y*-LP component. (**c**) The focusing performance of CM under forward *y*-LP incidence. The intensity distribution of the focal spot (i) on *x*-*z* plane at *y* = 0, (ii) along *z* axis when *x* = 0 and *y* = 0, (iii) on *x*-*y* plane at *z* = 30 μm and (iv) along *x* axis when *y* = 0 and *z* = 30 μm. (**d**) Far-field distribution of the reflection under backward *y*-LP incidence. (**e**) Transmittance comparison of CM and the single metasurface (M) mentioned above under *x*-LP incidence.

Figure 4a shows the schematic diagram of the functionality of the cascading metasurface (CM) for forward and backward incidences. It consists of an array of 14 × 50 supercells, having aluminum metal wire on the top (as shown in Figure 3a), a middle aluminum subwavelength grating, and an array of 50 × 70 bottom aluminum wire, wherein silicon dioxide spacers are sandwiched between two adjacent aluminum layers. The widths of all the aluminum wires are equal to obtain uniform phase distribution for *x*-LP component. To achieve focusing and deflecting functions, the lengths of the top and bottom wires were designed to satisfy the following phase distributions according to the corresponding relationship shown in Figure 2b, respectively.

(3) $$\begin{array}{l} \varphi_{\mathrm{Top}}(x,y)=\left\{ \begin{array}{l} -\frac{2\pi }{\lambda }\sqrt{f-(x^{2}+y^{2}+f^{2})}\begin{array}{cc} & x^{2}+y^{2}\leq D/2 \end{array}\\ \begin{array}{cc} 0 & x^{2}+y^{2}>D/2 \end{array} \end{array}\right.\\ \varphi_{\mathrm{Bottom}}(x,y)=\frac{2\pi }{\lambda }x\sin\gamma \end{array}$$

where *f* = 30 μm is the focal length of the design flat lens, *λ* = 1.55 μm is the incident wavelength, *D* = 30 μm is the diameter of the lens, and *γ* = 45° is the designed deflection angle.

The CM was simulated with the same method as mentioned above, but the perfectly matched layer boundary conditions were applied to *x*, *y*, and *z* directions. Figure 4c shows the simulated focusing spots of the proposed cascading metasurface for forward *y*-LP incidence at 1.55 μm. Results show that the focal point is at *z* = 30 μm, which is fully consistent with the design. The full width at half maximum (FWHM) of the focal spot is 1.98 μm, and it can be further compressed by increasing the diameter of the lens. Figure 4d shows the far-field distribution for the backward *y*-LP incidence. It can be found that the results of CM are the same as that of the single metasurface shown in Figure 3e, which implies that the two cascading cavities are functioning independently. The performances of the CM under *x*-LP incidences are shown in Figure 4e. Results show that the transmittance of *x*-LP components drops down to 0.72, while the transmittance of the *y*-LP components increases to 0.002 at 1.55 μm after cascading. This phenomenon may be related to the additional intrinsic absorption loss. Although the polarization selection performance deteriorates after cascading, the transmittance of the *x*-LP component is still higher than that of a single-layer grating, and the extinction ratio can reach 292, which can meet the needs of applications in which a high extinction ratio is not required. Moreover, the functionalities that can be achieved are not restricted to those presented. Hologram generator [10], vector beam generator [26], light sward [27], and other wavefront shaping functions could be achieved by imposing the required phase profile. In addition, although the metasurfaces demonstrated above were treated only theoretically, the fabrications of such metasurfaces are not difficult for the current micro-nano fabricated technology. For example, the metasurface can be obtained by layer-by-layer electron-beam lithography and coating processes. Owing to the robustness to misalignments of the top wire and grating wire, the requirement for the accuracy of the overlay is greatly reduced [28]. Therefore, besides the lithography method, the subwavelength grating can also be fabricated via the template-assisted lithography-free (colloidal self-assembly) methods, which is suitable for low-cost preparation of large-area periodic or quasiperiodic structures with size below 100 nm [29,30].

## 4. Conclusions

In this paper, an alternative approach to achieve the Janus metasurface is proposed based on a MIM metasurface. Comparing with the common MIM structure, the bottom metal mirror was replaced by a subwavelength grating with a polarization selection function. Therefore, for a forward incidence, the MIM structure can operate as a gap plasmonic cavity for the LP component parallel to the grating wires, and it acts as a highly efficient dual-layer grating polarizer for the orthogonal LP component, while for the backward incidence, the high reflection of the grating blocks the function of the gap plasmonic cavity, leading to its pure function as a polarizer. A bifunctional Janus metasurface for the polarizer and for the deflector was illustrated to prove the validity of the proposed metasurface. Simulated results show that for forward incidence, the proposed metasurface operates as a polarization filter for *x* polarization and as a 45° deflector for the y polarization; for backward incidence, it acts as an efficient polarizer with a transmission of 0.86 and extinction ratio of 3840 at 1.55 μm. Moreover, mutual influence is avoided since the functions corresponding to different propagation directions or polarizations are achieved using different operation mechanisms and dominated by different structure parameters. The benefit of this feature is that the proposed metasurface can be combined to achieve more flexible functions. A trifunctional Janus metasurface acting as a focusing lens, as a reflector, and as a polarizer is obtained based on this strategy. We believe that the proposed metasurface and the design strategy will provide more convenience in the design of multifunctional, miniaturized, and integrated optical components for various polarization-related applications.

## Supplementary Materials

The following are available online at https://www.mdpi.com/article/10.3390/nano11041034/s1, Figure S1: Simulated reflectance of (a) *y*-LP incident light and (b) transmittance spectra of *x*-LP incident light of the unit cells.
