# Supplementary material for: Design of Multifunctional Janus Metasurface Based on Subwavelength Grating
Source: Nanomaterials (Basel). 2021 Apr 19;11(4):1034. doi: 10.3390/nano11041034 (PMC8073647; doi:10.3390/nano11041034)
Supplement: Supplementary file 1 [file nanomaterials-11-01034-s001.zip › nanomaterials-1178858-SI.pdf]

nanomaterials

Supporting Information

Design of Multifunctional Janus Metasurface Based on Subwavelength Grating

Ruonan Ji [1,*], Chuan Jin [2], Kun Song [1], Shao-Wei Wang [3,*], and Xiaopeng Zhao [1]

1 Smart Materials Lab, School of Physical Science and Technology, Northwestern Polytechnical University, Xi'an 710129, China; songkun@nwpu.edu.cn (K.S.); xpzhao@nwpu.edu.cn (X.Z.)
2 State Key laboratory of Transient Optics and Photonics, Xi'an Institute of Optics and Precision Mechanics of CAS, Xi'an 710119, China; jinchuan@opt.ac.cn
3 State Key Laboratory for Infrared Physics, Shanghai Institute of Technical Physics, Chinese Academy of Sciences, Shanghai 200083, China
* Correspondence: jiruonan@nwpu.edu.cn (R.J.); wangshw@mail.sitp.ac.cn (S-W.W.)

The reflectance of y–LP incident light and transmittance spectra of x–LP incident light of the unit cells were simulated. As shown in Figure S1a, the resonant wavelength increases with the increase of the wire length. The reflectance at the wavelengths away from the resonant wavelengths can maintain above 0.8 in 1–2 μm. As for the transmittance of x–LP incident light, the increase in wire length has negligible impact on the transmittance spectra of x–LP incident light, but only the transmission peak redshifts from 1490 nm to 1560 when the wire length increases from 60 nm to 580 nm.

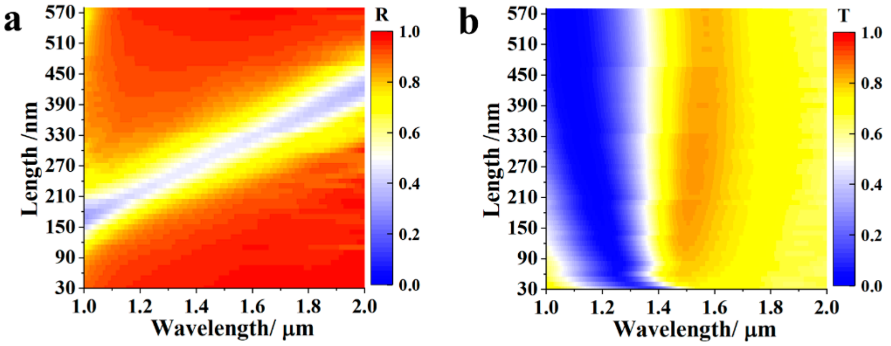

Figure S1 Simulated reflectance of (a) y–LP incident light and (b) transmittance spectra of x–LP incident light of the unit cells. The structure parameters were taken as p_x = 600 nm, p_y = 432 nm, w = 300 nm, w_g = 216 nm, and l varies from 30 nm to 580 nm.
